# Perceptions of food environments and nutrition among residents of the Flathead Indian Reservation

**DOI:** 10.1186/s12889-020-09584-7

**Published:** 2020-10-12

**Authors:** Carmen Byker Shanks, Selena Ahmed, Virgil Dupuis, Bailey Houghtaling, Mary Ann Running Crane, Mike Tryon, Mike Pierre

**Affiliations:** 1 Department of Health and Human Development, Montana State University, Bozeman, MT 59717 USA; 2 Food and Health Lab at Montana State University, Montana State University, 960 Technology Boulevard Room 245, Bozeman, MT 59718 USA; 3 Salish Kootenai College, Pablo, MT 59855 USA; 4 School of Nutrition and Food Sciences, Louisiana State University (LSU) & LSU Agricultural Center, Baton Rouge, LA 70803 USA; 5 Wellness/Group Fitness Program Supervisor, Kalispell Regional Healthcare, Kalispell, MT 59901 USA; 6 Food Department of Human Resources Development, The Confederated Salish and Kootenai Tribes, P.O. Box 278, Pablo, Montana 59855 USA

**Keywords:** Food environment, Dietary behaviors, Native American, Food system, Dietary quality, Food security

## Abstract

**Background:**

Indigenous food systems have been displaced with the emergence of colonization, industrialization, and cultural, economic, political, and environmental changes. This disruption can be seen in marked health and food environment disparities that contribute to high obesity and diabetes mellitus prevalence among Native American peoples.

**Methods:**

A Community-Based Participatory Research (CBPR) approach was used to document food environment experiences among residents of the Flathead Reservation in rural Montana. Participants were identified using purposive sampling techniques to participate in a survey and a semi-structured interview. Descriptive statistics helped to describe participant demographics, food access variables, and household food security status. Food environment perceptions were analyzed using the constant comparison method among trained researchers.

**Results:**

Participants completed surveys (*n* = 79) and interviews (*n* = 76). A large number participated in federal nutrition assistance programs. Many self-reported experiencing diet-related chronic diseases. Major themes included the community food environment, dietary norms, and food-health connections. Subthemes were represented by perceptions of food environment transitions and the important role of food in familial life. Further, opportunities and challenges were identified for improving community food environments.

**Conclusions:**

Perceptions of the food environment were linked to strategies that could be targeted to improve dietary quality along a social-ecological model continuum. There is need for skill-based education that directly addresses the time and monetary constraints that were commonly experienced by residents. Coinciding food environment interventions to promote dietary quality that engage community members, store management, and government policy stakeholders are also needed to reestablish healthy Native American food systems and environments within this community.

## Background

Across the globe, Indigenous food systems have been displaced with the emergence of colonization, industrialization, and cultural, economic, political, and environmental changes [1]. Indigenous food systems represent the cultural resources that Indigenous peoples possess about their local ecosystem to produce food for health and well-being. Indigenous people’s relationship with their food system contribute to their subsistence and food security, identity, spirituality, culture, language, history, and survival [1].

Across the United States (US) in the late 1700s, large scale seizures of tribal lands and movement of Indigenous peoples to reservations began dramatic shifts in Indigenous food systems [2, 3]. These events, paired with economic, political, and environmental changes, contributed to detrimental shifts in Indigenous food systems. Following, the food environments and diets of Native Americans has markedly transitioned from pre-contact to post-colonial times [3–11]. Thus, the US’ obstruction of Indigenous food systems, cultivated over thousands of years, has changed the trajectory of Native American livelihood and well-being.

The disruption of Indigenous food systems globally can be seen in the US as marked health and food environment disparities stemming from dietary change among Native American peoples. Herein, the term Indigenous refers to Indigenous people of the globe and the term Native American refers to Indigenous people of the US. Native Americans are 60% more likely to be obese than non-Hispanic whites and have a higher age-adjusted prevalence of diabetes mellitus than any other race or ethnic group in the US [12, 13]. Native Americans are more likely than the general US population to live in rural locations with limited food access in their food environment and experience low food security [14, 15].

Understanding food environments in Native American communities is key to restoring Indigenous food systems in the US and eliminating health disparities. The food environment influences the availability, affordability, quality, convenience, and desirability of food options that meet cultural and taste preferences as well as local standards of quality [16]. The food environment affects what people can and do eat and, as such, is an important upstream variable that can predict poor health outcomes including obesity [17]. Many Native American food environments of today exhibit the nutrition transition, which includes a shift towards the availability of foods high in sugar, fat, and processed foods [2].

Food environments include both built and natural spaces [16]. Built food environments are comprised of constructed places that provide food, including grocery stores, convenience stores, farmers markets, food assistance programs, restaurants, schools, and other retail outlets [18]. In the built food environment, Native American and rural communities face unique challenges to cultivating healthy diets, including limited infrastructure [19], long distances to food outlets [20, 21], lower quality [22–24] and less affordable foods [25] and overall fewer healthy options in their food environment [26]. Commodities, or the Food Distribution Program on Indian Reservations (FDPIR), was created to improve food access for reservation communities, but has been unsuccessful in providing a balanced diet or one that supports Native American food systems [27].

Further, the natural food environment consists of both wild and cultivated spaces, where food can be grown, hunted, or foraged, including fields, agricultural lands, forests, and home and community gardens [16]. Visible transition in natural food environments among Native American communities include the loss of tribal lands, shifting lifestyles from nomadic to settled, limitations on hunting, fishing, and wild food collection, and the endangerment of many food sources [2, 28].

Dietary patterns and nutrition-related disease vary and are shaped by culture, ecology, economics, politics, and technology, among other factors [29]. Community involvement in the research process integrates the populations and other stakeholders who the research involves and seeks to influence [30]. Researchers must complement a community’s priorities to provide recommendations and tools that will facilitate dietary change relative to current policies, systems, and environments. Collecting relevant data is essential to inform effective, community-specific food system projects to eliminate health disparities in Native American communities [1].

This study’s team included authors and a community advisory board that identified the need for a more in depth understanding about local perceptions of the food environment and diets among community members of the Confederated Salish and Kootenai Tribes of the Flathead Indian Reservation, remaining homeland of the Selis, Ksanka, and Qlipse People in order to restore the food system. Therefore, this research aimed to conduct a qualitative investigation about food environments and diets among Flathead Reservation residents, including tribal residents (i.e., members of the Flathead Nation) and non-tribal residents to inform programs, policy, and practice around food and nutrition in the future.

## Methods

### Study setting

The Flathead Reservation of the Confederated Salish & Kootenai Tribes (CSKT) in rural northwest Montana is territory of the Bitterroot Salish, Kootenai, and Pend d’Oreilles tribes. Arlee, Saint Ignatius (Mission), Hot Springs, Ronan, Pablo, Polson, Elmo, and Charlo are recognized townships of the Flathead Reservation, with other small outlying communities present. In total, 28,993 people resided on the Flathead Reservation in 2015, with approximately 5000 enrolled tribal residents living on or near the reservation of 7791 enrolled tribal members [31, 32]. A majority of other residents identify as White or Native Americans that are enrolled members of other tribes. The Flathead Reservation is 1938 mile^2^ and the distance between towns ranges between approximately 9 miles to over 60 miles. Montana is classified as a frontier and remote state and is also the most rural state in the United States by percentage of residents residing in rural areas [33, 34]. Some individuals reside in more densely populated areas of townships, where others reside in smaller and more rural communities.

There are a high proportion of limited income households with low levels of food access [35]. During data collection, the Flathead Reservation had 13 grocery stores. Participants of this study also regularly accessed foods from convenience stores. Restaurants and fast food outlets were mostly concentrated in more populated townships described above. Agricultural lands with crops and livestock in production make up a portion of the lands, including fruits and vegetable, cattle, and grains. Much of the agricultural production in existence is not traditional to the CSKT and have presented in modern times.

Wild foods are present across the Flathead Reservation and are accessible via the natural food environment, including near some residents’ homes and in areas that require transportation to access hunting, gathering, and fishing areas. Varying access exists, given that the Flathead Reservation is nearly 2000 miles squared, the places that residents reside within minutes or hours of wild food, and lawful licensing is required for non-tribal members. Flathead Lake is the largest freshwater lake in the US located to the northeast side of the Flathead Reservation. The Flathead River also flows through many parts of the Flathead Reservation and into Flathead Lake from the southwest. Both bodies of water provide opportunities for fishing.

The following food assistance programs commonly support Flathead Reservation residents, including the Supplemental Nutrition Assistance Program, Food Distribution Program on Indian Reservations (FDPIR), National School Lunch Program (NSLP), and Special Supplemental Program for Women, Infants, and Children (WIC), among others. Approximately 15 % of residents are food insecure across Lake County, which includes both tribal residents as well as non-tribal residents [36].

### Approach

Community-Based Participatory Research (CBPR) methods [29] guided each stage of the research process. The authors include tribal community members of the Flathead Reservation, researchers affiliated with Salish Kootenai College, and non-tribal researchers from Montana State University that participated in all aspects of the research process. To ensure a culturally relevant approach, the authors collaborated with a community advisory board of food and nutrition stakeholders at the Flathead Reservation in order to decide upon the direction of the research at the onset. The research focus was decided as little was known about the experiences and perceptions of the food environment among residents of the Flathead Reservation in rural Montana. Residents of the Flathead Reservation include both individuals that are enrolled tribal members and non-tribal members. Tribal refers to only enrolled citizens of the Confederated Salish and Kootenai Tribes. Non-tribal refers to individuals living on the Flathead Reservation and are not enrolled citizens of the Confederated Salish and Kootenai Tribes. This research gained approval by both the Montana State University and the Salish Kootenai College Institutional Review Boards for research with human subjects. All participants provided written consent to participate before data collection commenced.

A qualitative approach using interviews and surveying was deemed appropriate for gathering knowledge from residents of the Flathead Reservation. The long-term goal was to use the results to inform programs, policy, and practice around food and nutrition in the future. The community advisory board was consulted in a series of meetings and communications to contribute to the research questions and protocol, which was amended based upon input. The community advisory board then participated in the interpretation of the resulting data by reading through anonymous results, suggesting themes, and organizing themes and subthemes during. Additionally, the results were presented at The Confederated Salish and Kootenai Tribes’ Tribal Council for input from members that represent all districts of the Flathead Reservation. Using a CBPR approach across the research process privileged the local ecological knowledge of residents of the Flathead Reservation over Western science perspectives.

### Role of researchers

The research team was a mixture of outside, non-tribal researchers (*n* = 4) and tribal (n = 4) and non-tribal members (*n* = 2) of the field site community. The researchers developed a qualitative data collection protocol, with standardized procedures and probes, and trained together practicing various survey and interview scenarios until all team members felt comfortable leading one-on-one interviews, which took approximately 2 hours. Involved partners included community stakeholders, educators, students, and Tribal Council within the tribal community to provide input and contextualize research data for academics and students within the partnered university communities.

### Recruitment

Purposeful sampling was used to recruit participants who were household decision-makers in eight recognized townships within the bounds of the Flathead Reservation to understand how the broad food environment impacts both household dietary quality and food security. Members of the research team that were local residents and the community advisory board helped to recruit Flathead Reservation residents through a snowball sampling approach from the townships. The goal was to recruit a sample that represented diverse demographics in order to attain a deep understanding of food environment and diet perspectives among Flathead Reservation tribal and non-tribal residents. A tribal resident is an individual that is officially enrolled as a member of one of the local tribes, with criteria for tribal enrollment being different across reservations. A total of 80 participants were recruited for surveys and interviews; however, four were unable to complete the interview portion due to time constraints with caring for families and employment. No eligible participants declined involvement. Participation in both portions of the study resulted in an incentive valued at $50 USD.

### Data collection

Data was collected with semi-structured interviews and sociodemographic surveys between May and August of 2015. Tribal and non-tribal researchers collected data face-to-face and one-on-one with each participant. The researchers answered questions when needed during the survey completion and facilitated dialogue during the audio-recorded interview process. Survey information were collected in a paper form and included demographic information, typical modes of household food access, and responses to questions probing food security status (USDA’s Six-item Short Form Food Security Survey Module) [37]. Interview questions were developed by reviewing the literature around perceptions of the food environment and nutrition, food systems broadly, and food systems specific to indigenous communities (Table 1). The community advisory board reviewed and provided input on the questions. Interview questions were asked in a dialogue format and focused on dietary intake, nutrition knowledge, food access in the food environment, and perceptions of the community food environment and diets. The semi-structured interview style allowed the interviewer to use standardized probes for further responses and follow conversation trajectories where appropriate under the interview protocol. The interview and survey took under 1 h to complete, with a range of 40–60 min for participants.

**Table 1:** Qualitative Interview Questions Used to Understand Participant Relationship with Community Food Environment of the Flathead Reservation

*Section One: Dietary Intake*	
• What are some of the most popular foods eaten in your household?	
• What are some of the most popular foods eaten in your community?	
• Do any of these foods have cultural value to your community?	
• How often do you prepare meals at home?	
• How often do you eat out?	
*Section Two: Nutrition Knowledge*	
• Do you know any foods that you should not eat too much of?	
• Why should you not eat too much of these foods?	
• What is your idea of a healthy diet?	
• What motivates you to eat healthy?	
• What prevents you from eating healthy?	
• Where did you learn most of your information about food?	
• In what ways is it easy or hard to get your family to eat healthy foods?	
• How do you respond to resistance from family?	
*Section Three: Food Access in the Food Environment*	
• What are all the sources of food in your community?	
• Do you or someone in your household hunt?	
• Do you or someone in your household fish?	
• Do you or someone in your household collect wild foods?	
• Do you have a home garden?	
• Would you be interested in learning any techniques of how to grow food and take care of a home garden?	
• Do you participate in food assistance programs? If so, what do you like or not like about these programs?	
*Section Four: Perceptions of the Community Food Environment and Diets*	
• Have the foods that you eat in your household and community changed in the past decade? During your lifetime? If so, what are some of the reasons that these foods have changed?	
• Do you feel that general diets in your community at present are healthier, less healthy, or the same as in the past?	
• For people who live in your area who cannot get enough food, or who have difficulty getting enough food, what resources are available to help them out?	
• If you could be in charge of improving eating habits for your community, what changes would you want to make from the way things are now?	
• What are the main obstacles to achieving this goal?	

### Data analysis

Quantitative survey information was de-identified and coded within a master list respective to question responses. Descriptive statistics were utilized in order to determine means, standard deviations, and simple percentages dependent on response type to describe participant demographics, food access variables, and to understand household food security status.

For the qualitative data, thematic analysis was conducted guided by the constant comparison [38]. Interviews were first de-identified and then transcribed. Researchers then differentiated transcribed text into smaller units, which are independent thoughts within a participant’s statement [38], resulting in 3814 units. Next, codes were created in a draft codebook by tribal and non-tribal researchers based on a random sampling of units. The draft codebook along with sample units was presented to the community advisory board for input and revised based upon recommendations. Using the constant comparison method, the research team members compared each code and unit to the existing findings for similarities and differences in the data throughout the process. All interview transcripts were then coded separately by two team members and compared. All discrepancies in coding (*n* = 2209) were ultimately resolved between coders. Thereafter, coded meaning were sorted based on question type. All researchers collaborated to describe and organize thematic outcomes of interview data. A total of 3712 units contributed to themes, after non-relevant units were removed (*n* = 102). Researchers presented the data to their community advisory board and Tribal Council to provide additional perspective. Conversations about the data surrounded the social ecological framework and is presented as such in the discussion section of this manuscript.

## Results

Theme frequencies contributed by participants are presented in Table 2.

**Table 2:** Frequency of Participant Responses Involved in Qualitative Food Environment Interviews in a Native American Community

Major Themes	Subthemes	Participants Contributing (Number of Units^a^)
(1) Description of the Community Food Environment	(1.1) Food Assistance Contribution to Food Security	76 (1067)
		76 (601)
(2) Dietary Norms	(2.1) Food Environment Transitions	76 (489)
	(2.2) Food and Family	68 (271)
(3) Food-Health Connection		75 (687)
	(3.1) Opportunities and Challenges for the Community Food Environment	76 (597)

^a^One unit is an independent thought within a participant’s statement [38]

### Socio-demographics

A total of 76 participants were interviewed and 79 participants completed surveys. Participant demographic information is available in Table 3. A majority (73.4%) reported being Native American and 50.7% grew up on the Flathead Reservation. Many (*n* = 38) reported household prevalence of one or more diet-related non-communicable diseases including obesity (73.7%), diabetes (50.0%), and/or cardiovascular conditions (7.9%).

**Table 3:** Descriptive Statistics Regarding Demographic Characteristics of Interview Participants Living on the Flathead Reservation, *N* = 80

Category	No. Respondents	%	Mean (SD)
**Age**	79		40 (11.6)
**Sex**	80		
Female		78.75	
Male		21.25	
**Education – High School**	77		
*Partial*		18.2	
*Graduate*		81.8	
**Education – College**	76		
*No College*		21.1	
*Partial*		39.5	
*Graduate*		39.5	
**Race/Ethnicity**^a^	79		
*African American*		5.1	
*Native American*		73.4	
*Hispanic*		2.5	
*White*		29.1	
**Marital Status**	61		
*Single, Never Married*		26.2	
*Unmarried, Cohabitating*		23.0	
*Married*		42.6	
*Divorced or Separated*		6.6	
*Widowed*		1.6	
**Native Country**	80		
*United States*		98.8	
*Other*		1.3	
**Childhood Community**	77		
*On Reservation*		50.7	
*Neighboring Reservation*		23.4	
*Off Reservation*		26.0	
**Years in Community**	80		24 (17.0)
**Farming Childhood**	79		
*Yes*		19.0	
*No*		81.0	
**Household Size**	80		
*Adults*			2 (1.1)
*Children*			3 (1.4)
**Age – Boys**	61		10 (7.0)
*Less than 1 year*^c^		5.3	
**Age – Girls**	60		10 (6.9)
*Less than 1 year*^b^		2.2	
**Household Income**	80		
*Less than 15 K*		30.0	
*15,001 K–25 K*		21.3	
*25,001 K–35 K*		20.0	
*35,001 K–50 K*		15.0	
*More than 50,001 K*		13.8	

^a^Some participants identified as multiple races/ethnicities (*n* = 7)

^b^Percentage out of total reported female children (*n* = 92)

^c^Percentage out of total reported male children (*n* = 95)

Percentages within a category may not add up to 100% due to rounding

Food access sites in addition to shopping and traveling behavior are displayed in Table 4. Most reported owning a vehicle (*n* = 72) and stated that groceries were mainly purchased (*n* = 74), although many reported transportation an issue due to long commute times and car sharing. Utilities to support food storage were prevalent in the sample, evidenced by having a standard size refrigerator, (*n* = 75) stand-alone freezer unit (*n* = 46) or, less frequently, a small refrigerator unit (*n* = 8). Participation in government assistance programs was common among research participants (Table 4).

**Table 4:** Descriptive Statistics Regarding Food Access of Interview Participants Living on the Flathead Reservation, *N* = 80

Category	No. Respondents	%	Mean (SD)
**Source of Transportation**^a^	44		
*Self*		61.4	
*Family*		11.4	
*Friends*		2.3	
*Spouse*		11.4	
*Public Transport*		13.6	
*Taxi*		2.3	
**Nutrition Assistance Program Participation**^a^	54		
*SNAP*		51.9	
*FDPIR*		5.6	
*WIC*		27.8	
*Reduced, Free School Lunch*		48.2	
*Free Breakfast for Children*		16.7	
*Other*		13.0	
**Primary Food Access Site(s)**^a^	78		
*Grocery Store*		76.9	
*Family or Friends*		1.3	
*Local Food*		1.3	
*Small Food Store*		1.3	
*Specialty Food Store*		7.7	
*Supercenter*		37.2	
*Unknown*		6.4	
*Wholesale Store*		15.4	
**Secondary Food Access Sites(s)**^a^	62		
*Convenience Store*		1.6	
*Department Store*		4.8	
*Grocery Store*		43.6	
*Family or Friends*		4.8	
*Food Assistance Programs*		3.2	
*Local Food*		11.3	
*Small Food Store*		9.7	
*Specialty Food Store*		11.3	
*Supercenter*		46.8	
*Wholesale Store*		17.7	
**Travel Distance to Food Access Sites**^a^	61		
*Miles*			25 (23.0)
*Less Than One Mile*		3.3	
*Minutes*	65		50 (48.8)
**Typical Travel Mode for Grocery Shopping**^a^	80		
*Walk*		7.5	
*Personal Car*		87.5	
*Ride with Family or Friends*		11.3	
*Taxi*		2.5	
**Frequency of Grocery Shopping**^a^	80		
*Once Per Week*		73.8	
*Every Two Weeks*		20.0	
*Once a Month*		10.0	
**Primary Shopper**^a^	79		
*Self*		90.0	
*Spouse or Partner*		25.3	
*Other Family Member*		10.1	
**Typical Grocery Expenditure ($)**^a^	80		
*< 50/week*		2.5	
*50–99/week*		30.0	
*100–199/week*		31.3	
*250/month*		7.5	
*350 month*		21.3	
*Other*		8.8	
**Standard or Small Refrigerator**	80		
*Yes*		2.5	
*No*		97.5	
**Government Assistance Services Utilized or Applied For**^a^	30		
*Transportation Assistance*		13.3	
*Food Bank*		40.0	
*Home Health*		3.3	
*Homemaker Services*		3.3	
*Housing/Rental Assistance*		30.0	
*Nutrition Counseling*		3.3	
*LIEAP*		53.3	
*Other*		6.7	

^a^Participant(s) responded with more than 1 choice

Percentages within a category may not add up to 100% due to rounding

Food security, or the access to enough and appropriate foods for an active and healthy lifestyle, is a major concern among the participants. Approxamitely 50.0% of participants (*n* = 40) reported low or very low food security status, while the remainder scored high or marginal food security (*n* = 39).

### Thematic analysis

Three overarching themes emerged from the interview process. Theme 1, Community Food Environment, described participant reliance on both the built and natural environment for food acquisition; Theme 2, Dietary Norms, illustrated perceptions of popular or common foods in both the household and community, and; Theme 3, Food-Health Connection, captured current nutrition knowledge and participant perceptions about healthy eating patterns.

Study subthemes provide more information to support themes. Subtheme 1.1, Food Assistance Contribution to Food Security, highlighted the reliance and utility of both federal and community food assistance programs within this community, making it an important component of the community food environment. Subtheme 2.1, Food Environment Transitions, included participant descriptions of the cultural value of common foods as well as perceived changes in the food supply over time that informed dietary norms. Additionally, subtheme 2.2, Food and Family, focused on the influence of family on food norms and health behaviors. Last, subtheme 3.1 articulated opportunities and challenges for improving the community food environment to improve health outcomes from participant perspectives.

### Thematic results

#### Theme 1: community food environment

Both the natural and the built environment were important sources of food access within the study population. Most often, grocery type stores were mentioned as key food access points in the built environment. Though, restaurants and convenience stores were also a source of food as detailed by participants, especially under time (e.g., caring for families, multiple jobs) and money constraints. As stated by one participant, “*[Fast food restaurant chain] or [Fast food restaurant chain], that’s about the extent of it [ …*] *on a rare occasion it’s [Fast food restaurant chain], but that’s expensive for six of us so [ …*] *we try to go something where it’s cheap but it’ll get everybody fed (P8)*.” Within the natural environment hunting, fishing, and collecting wild foods were common in addition to utilizing home or community gardens. The farmer’s market was listed less frequently, perhaps due to its seasonality, and is one example of a connection between the two environmental sources of food, “*With the farmers markets starting up, we do a lot of that (P7)*.”

Interview participants described that food access was one barrier to acquiring food for their families. A lack of reliable transportation or funds to support long distance travel to access a variety of foods were commonly described by participants. For example, “*They don’t have a car or, if they do have a car, they don’t have a truck to go into the mountains to get fresh berries and things like that (P23)*.” Further barriers to food access were described as a lack of food availability and desirable options, “*Nothings organic, everything’s non-organic, vegetables and fruit, they don’t have fresh sometimes, it’s like kinda rotten and then they don’t really have meat too much there and if they do its all processed meats, so hot dogs and lunch meats [ …*] *and they have a lot of pop, they have a ridiculous amount of pop (P17).”* Other participants described a total lack of food stores near their residence, “*We have no sources of food in our community (P53)*.” Often this prompted participants to travel further distances for food needs (Table 4).

Within the natural environment, hunting, collecting wild foods, and home gardens were common modes of accessing food exterior to the built environment. Overall, huckleberries, chokecherries, service berries, bitter root and a variety of meat sources including buffalo, deer, fish, and elk were commonly harvested. Often such activities were described as a family or community affair. For example, sharing of these food sources were common amongst extended members outside of the household, “*Like if we’re lacking like huckleberries or something, like if we’re lacking meat, all we need to do is just go and ask one of the family members and then they give it to us even in our community (P23)*.” Few participants mentioned access to wild foods/natural environment was not available to them, “*No I don’t have a hunter or gatherer (P30)*.”

Gardens were a popular point of discussion point for many participants with varied methods utilized, ranging from small “*Right now my daughter and I are trying to do our own garden. We’re doing a bucket garden (P18)*,” to full home plots, “*My garden is 50’ by 80’ it’s huge (P72)*.” A small number of participants described perseverance in cultivating a garden despite barriers, “*This is my third garden. We will see how it goes. I tried, I totally have not given up. If it don’t work this time, I may give up (P46)*” and many more were interested in learning gardening strategies. A barrier to gardening, stated most often, was a lack of knowledge or available space, because of a small yard or restrictions from local authorities, and time, due to jobs and family activities. For example, in response to questions to determine gardening capacity and interest, responses included: “*I wanted a home garden but don’t have time and don’t know how (P63)*,” and; “*No room (P2)*.”

In lieu of a personal gardening plot, participants expressed that community gardens were another mode for accessing fresh produce, especially for those facing food access barriers. Seven community gardens were available within the field site community and also helped to provide foods for elders, “*A lot of our seniors [ …*] *were raised in [ …*] *poorer times and had gardens, but now that they’re older it’s hard for them to have their own garden and grow that, so it’s good for them to get fresh um, non- chemical treated produce to take home and some of the older ladies still love to can (P23)*.”

#### Theme 2: dietary norms

Fruits and vegetables were more reported as common food items available and consumed within households. However, interview participants perceived that produce was not a common food item available and consumed within other households in the community. Fruits, including strawberries, apples, and bananas, were reported more frequently than vegetables (broccoli was the most commonly mentioned in this category). Ultra-processed or fast food products were reported both as more available and consumed within other households in the community than within the interview participant’s household. Further, protein sources such as beef in the form of hamburger and starchy foods such as potatoes and pasta were often reported as popular food items both within household and community domains.

Table 5 provides information specific to participant perceptions of common food items acquired from the built food environment and then consumed within the household and the community. Most families consumed food away from home and also prepared food at home. Most participants stated that fast food or community restaurants were most regularly visited when eating food away from home. A smaller number of participants noted convenience and money as a large factor in deciding between food away from home and food at home. For example, on participant stated, “*We usually don’t go out to eat but this past month, it’s been so busy that I would get home at 7 or 8 and be too tired to cook, so then like we’d go and eat out, which is really bad, cause it really hurt our budget (P11)*.”

**Table 5:** Perceptions of Frequently Accessed Food Items Within the Household and Within the Community From the Built Food Environment on the Flathead Reservation, Organized by Prevalence, *N* = 77^a^

Within Household	% Participant Responses	Within Community	% Participant Responses
Beef	40.3	Hamburgers	22.1
Vegetables	39.0	Meat	22.1
Fruits	38.0	Pasta	20.8
Pasta	26.0	Fry Bread	16.9
Potatoes	22.4	Potatoes	16.9
Chicken	22.1	Fast Food	15.6
Cereal	19.5	Wild Game	14.3
Game Meat	18.2	Bread	13.0
Pork	13.0	Pizza	13.0
Pizza	11.7	Soda	10.4
Fish	11.7	Processed Foods^b^	10.4
Tacos	10.4	Chips	10.4
Bread	10.4	Hot Dogs	9.1
Chips	9.1	Desserts	9.1
Yogurt	9.1	Fruits	7.8
Eggs	9.1	Vegetables	7.8
Salad	9.1	Greasy/Fried Foods	7.8
Milk	9.1	Indian Tacos	6.5
Sandwiches	7.8	Salad	6.5
Popcorn	7.8	Eggs	5.2
Desserts	6.5	Barbeque	5.2
Rice	5.2		
Water	5.2		
Cheese	5.2		

^a^Foods were only included in this table if mentioned by at least 4 (~ 5%) participants

^b^The participants described “processed food” as a broad category of unhealthy foods

Table 6 provides information specific to participant perceptions of common food items from the natural food environment. Fish, deer, elk, berries, bison, and roots were reported most commonly as popular food items within household and community domains from the natural food environment.

**Table 6:** Food Items Frequently Accessed from the Natural Food Environment as shared in Interviews on the Flathead Reservation, *N* = 77

Within Natural Environment	% Participant Responses
Fish	37.7
Deer	32.5
Elk	26.0
Berries	20.8
Bison	13.0
Roots	6.5

Foods were only included in this table if mentioned by at least 4 (~ 5%) participants

#### Theme 3: food-health connection

Participants stated that they learned about the connection between food and health in a variety of ways that informed dietary habits and health beliefs. For example, one participant explained learning about food and health knowledge, “*Just growin’ up in the community I guess (P18)*.” Others learned about food and health knowledge from a medical professional educating about a diet-related diagnosis, *“Just so from pre-diabetic and recently being told I’m a diabetic (P65)*.” Additionally, popular media avenues also played a role in gaining food and health knowledge, as stated by one interviewee, “*Reading it and google it you know (P62)*.” Also, schools were described as a source of nutrition and physical activity lessons to children during the day, “*My oldest boy [ …*] *he’s like picked up on a lot, like what’s healthy, what’s not, [ …*] *as far as fitness-wise, what he should be doing [ …*] *instead of sittin’ at home in front of a video game (P10)*.”

Fruits and vegetables were frequently mentioned as foods that contributed to a healthful diet, “*Some greens more colorful things, you want your plate to be colorful, [ …*] *not just meat and potatoes (P74)*,” as well as the importance of hydration, “*Drinking a lot more water (P50).*” Participants also described a connection with the natural environment, i.e., consuming wild game or berries, as indicative of a healthy diet, “*We still eat some of the native food, we pick berries every spring, we serve native soups well like we make pemican, dry meat, dry buffalo, jerk meat, venison (P43)*.” Portion control and meal timing were two commonly reported dietary habits, “*Healthy proportions, to know how much you are eating (P55)*,” and, “*Three meals a day, at proper times (P19)*.” Albeit more infrequently, physical activity was another component that informed healthy habits, “*Tryin’ to get your family outside is important to us (P55)*.”

Many participants confidently described a healthy diet, but a subset seemed unsure of how to describe components of a healthy diet, “*I really don’t have an idea [ …*] *because the question is what is healthy (P75)?*” One participant mentioned specific nutrients to avoid, *“Either high in sugar, high in sodium, high in fats (P12)*.” Further, high starch or simple carbohydrate foods were also frequently identified within this context, “*I would say potatoes, macaroni, and bread which we eat a lot of all of them (P22)*.” Taste was also a noted factor in choosing unhealthy’ items, “*Bad for you, but tastes good (P20)*.” Participants also shared the perceived importance of organic products. For example, one participant mentioned how her family should be practicing health by, “*Eating healthy and choosing to buy organic food and growing our food (P16)*.”

In a few cases, participants described nutrition knowledge that is inconsistent with what was described as a healthy diet by the 2015–2020 Dietary Guidelines for Americans (DGA) [30]. For example, one participant mentioned legumes as a food to be limited, “*Beans, I don’t know (P46)*.” Fish and fruits were also identified as necessary to limit in the diet, “*Fish, not supposed to eat too much fish (P37)*,” and, “*If you eat too much grapes [ …*] *you can get an upset stomach or kids can [ …*] *fruits, I don’t know (P9)*.” Also, as stated by another participant, “*Whole wheat is probably the worst thing you should eat in bread because it has a high sugar (P39)*.” Additionally, alternative products such as sugar-free or certain brands were termed as healthful, in comparison to usual purchases, “*Nutri-grain bars [ …*] *but that’s super expensive, so like can’t afford that then we’ll just buy the regular granola bars [ …*] *We eat a lot of pop tarts, but like I try to make them get like the sugar-free ones (P11).*” Limiting ultra-processed foods was perceived to be beneficial for many, however, one participant seemed unsure of this association, “*[ …*] *Those Lunchables, cause I think they’re healthy but I know that they’re processed (P12)*.”

Incentive to avoid unhealthy foods were primarily about participant concerns of developing diet-related non-communicable diseases, “*Bad health, obesity, clogged arteries (P69)*,” and “*Sugar is bad for you [ …*] *it causes diabetes and become overweight (P46)*.” Many participants described wanting to practice positive health behaviors to assure quality of life and longevity for themselves and their families, “*I want to be around for my grandchildren for a long time (P64)*.” Other concerns of poor dietary habits that were mentioned included feeling poorly, “*Not sleeping too well no energy (P66)*,” or dental issues, “*It’s not healthy for you. It rots your teeth (P71)*.” A few participants linked the deviation from traditional foods over time to the poor health outcomes experienced today, “*Indian peoples [ …*] *are only a couple generations away from their [ …*] *ancestral diet, [ …*] *it’s like harder for our bodies to [ …*] *digest that stuff [ …*] *and [ …*] *leads to diabetes (P17)*.” Another described the relationship between poor dietary choices and a decline in overall global health, “*Our mental, physical, emotional, spiritual, holistic wellness I think is the most important reason not to, because those foods do effect most parts of our life (P16)*.”

### Subthemes

#### Subtheme 1.1: food assistance contribution to food security

Government food assistance and community food programs also helped increase food security for participants and their families. SNAP and WIC were the most commonly discussed regarding program participation and many used words such as “*Helpful (P79)*,” “*Wonderful (P6)*,” and “*Good value (P57)*” to describe them. However, these perceptions might not have represented everyone’s beliefs, “*There are ups and downs, some people don’t like it [ …*] *other people are ok with it (P62)*.” At times overcoming negative perceptions of participating in food assistance programs was mentioned, “*We’re talking about being too proud […*] *I tell them that I’m on food stamps you know, and I’m a coach and I don’t make enough money […]and, so whatever [people] may think or believe, we don’t feel that way, cause, I think we, we’re buying some good food with it (P5).*

A lack of money for adequate foods was a main reason food assistance programs were utilized, as summarized by a member of a four-person household, “*My husband works minimum wage. I work very minimum wage [ …*] *and that is not enough to feed your family (P15).”* Participants also noted that provided food resources were not sufficient, “*We are pretty much out of groceries at the end of the month (P58)*.” At times household income prevented enrolling in programs with income eligibility cutoffs, “*They said I made too much money (P2)*.” However, despite this food assistance participation was desirable to help make ends meet, “*If I could I would definitely be on food stamps (P61).”*

At times the food products offered by various assistance program benefits were discussed, either negatively or positively. For example, a WIC recipient expressed a lack of preferred foods versus an abundance of non-preferred foods “*I only got like a $10 limit on my fruit and vegetables because they were higher priced. Like we just went through them really fast. [ …*] *I felt like I had 20 cans of peanut butter, like we really never used that much peanut butter (P44).”* Another interviewee mentioned the utility of SNAP’s seed distribution program along with a perceived lack of community knowledge, “*I think they should advertise that more (P8)*.” Interestingly, there were differing perceptions of the WIC food package provisions on participant dietary patterns. For example, it was stated that participation in WIC helped to promote healthy eating, “*If given the choice I wouldn’t eat healthy [ …*] *With WIC I have to eat healthy [ …*] *I have to eat what they give (P27).”* Contrary, another described the perceived unhealthfulness of offered foods through WIC, “*With WIC they’ll have certain items that you can’t get, which might be the most healthiest item (P69)*.”

Food banks or pantries and commodities (FDPIR) were also prevalently discussed as major sources of food for families. One participant liked the social atmosphere at the local food bank, “*They’re really good and sweet to us (P71).*” However, some mentioned a lack of availability, “*The food bank don’t have enough to give us you know (P58),”* or inadequate communication regarding service times as barriers, “*Even when I was really strugglin’ I never went to the food bank [ …*] *you don’t know what times (P49)*.” Commodity (FDPIR) programs were favorably regarded and also perceived to offer healthier foods now than the program did in the past, “*A lot healthier than what it used to be. Used to be, what cheese, the American cheese, some butter, and some canned meat (P54)*.” However, one participant mentioned the lack of reliability of the commodity food offerings, “*They have buffalo. By the time I get there, there is no fresh veggies and stuff (P64)*.”

#### Subtheme 2.1: food environment transitions

Some participants shared examples of changes to the local or global food environments over time, while others did not have knowledge about any transitions. Changes specific to perceived healthier types of foods available now were mentioned frequently, “*Over my lifetime when I was growing up we didn’t get much fresh like canned. Like with my kid I try to get it fresh you know (P24).”* Organic foods were also mentioned in this capacity, “*Bigger push for organic food, for uh, kind of healthier eating (P47).*” A few noted that health is a larger concern among community members today or, i.e., the demand for healthful foods had increased, “*I’ve made [ …*] *effort to, to make changes for my health (P53).*”

For those who believed diets are less healthy today, food costs and monetary barriers in the food environment are considered more pronounced, “*The price of food, the price just of the basic, of like staples (P50)*.” Time was another constraint when making food choices in today’s society, “*I think that people are always in a hurry these days (P35)*.” Overall, a lack of time was associated with a greater reliance on fast foods, equating to less healthier diets. Others believe that food preservation techniques of the food industry were less healthy, “*Things that they put in the meats to keep them looking nice in the store (P22)*,” and “*you have to watch now the pesticide that they are spraying on the crops (P25)*.

Others shared examples of varying modes of food access by time period. For example, traditional food access was linked with the land and therefore healthier, “*In that past the diets were extremely healthy, because [ …*] *everyone was living off the, the land literally and [ …*] *so there wasn’t processed food (P16)*.” Then after periods of assimilation and allocation of Tribes on reservations, food availability changed dramatically, “*Major poverty and um government is giving you rations. You know we’ve all heard stories about you know maggots in the rations that their giving to Natives (P16)*.” These events were described as influential on current perceptions of food and culture within the community. For example, most referred to Indian tacos and fry bread as cultural staples, “*That’s kind of one of your identities of being a good Indian woman in a good provider and a good cook is how good of a fry bread maker you are (P23)*.” While some acknowledged the influence of past tragedies on current cultural perceptions around food, “*We really didn’t get fry bread until after you know, the commodities come and you know, we were like, they are giving us flour you know. We didn’t eat flour so it’s not a traditional food (P45)*.”

Additionally, many of the common foods sourced from the natural food environment, a traditional mode of food access, were also considered to have cultural value. However, a portion of participants did not perceive that foods within in the community were culturally valued. Rather, as one participant mentioned, it is more about community and socializing, “*No, people get together and have a good time and be happy (P46)*.

#### Subtheme 2.2: food and family

Family was a major source of food knowledge. Many talked of learning from mothers and grandmothers as they grew up, “*It was just mainly the way I was raised with my parents (P23)*.” Food practices used to promote healthful options within the familial household seemed to be well received, “*Whatever I cook, they eat, so they pretty good about it (P33)*.” In encouraging healthy eating habits, participants had differing views and ways of encouraging family members to try foods. The most commonly mentioned method as stated by one participant, was meal consistency, *“I don’t let them eat a separate meal (P50)*.” However, another participant stated, “*I just give in and give them whatever (P70)*.” Time and money were commonly mentioned as barriers to promoting healthy eating practices within families. Another barrier was the perceived poor taste of healthful foods, “*Some of the healthier foods isn’t as sweet and good tastin’ to kids (P52)*.” Others mentioned that they themselves had not grown up in an environment where healthy foods were available or encouraged, making it difficult to change, “*It’s hard because we were brought up on meat, potatoes, and bread (P25)*.”

#### Subtheme 3.1: opportunities and challenges for the community food environment

Limited monetary resources and time were again reported as the main reason for why healthy dietary patterns were difficult to obtain. Additionally, poor food access was also a barrier to a healthy diet, albeit described less frequently in this context. Participants mentioned that taste perceptions were also a barrier to incorporating healthful foods, “*Just like the other stuff better still (P56)*,” and described a need for education, “*Maybe lack of knowledge on how to prepare food or what type of foods to buy (P35)*.” Generating enthusiasm to change lifestyles was also considered to be a barrier, “*Like I see lots of things [programs] out there, but it’s just people’s reluctance to, to move forward (P55)*.”

Building skills and capacity for gardening was a welcomed idea for most participants who stated interest and a need. At times, people mentioned disinterest due to a lack of time, “*I would be interested but my job sometimes I get home at 7 or 8 in the evening. It takes most of my day (P29)*.” Overall, promoting healthier eating habits was considered needed due to diet-related non-communicable disease prevalence in the community, “*Because the obesity and diabetes alone here in this community is horrible (P39).*” Interventions or methods to improve access to fresh foods by lowering costs would also be beneficial to achieving this, “*Makin’ fruits and vegetables easier and readily available at a lower cost probably (P52)*.” More opportunities for convenient, healthful options was also mentioned as a potential method to promote health, “*More business establishments encouraged to offer healthy food [ …*] *that was open, evening and then the early, early mornings (P17).*” Further, cooking classes that covered a wide range of topics such as canning, food safety and storage, quick preparation, and healthy recipes were other ideas mentioned by participants for needed changes within the community.

## Discussion

In this rural Native American community, both built and natural food environments were important sources for accessing food. However, there were multi-sectored [29] barriers to accessing and consuming foods and beverages aligned with the 2015–2020 DGA described by community members of the Flathead Reservation. As such, a socioecological model is used as a framework to contextualize thematic results and highlight opportunities for enhancing dietary quality among Flathead Reservation communities [29]. Individual knowledge, attitudes, beliefs, skills, resources, and social networks, in addition to policy, systems, and environmental (PSE) factors, need be aligned to establish healthy dietary patterns and promote health equity. Although presented linearly, multi-sectored actors described below indicate the value of qualitative data to inform multi-level changes individual behaviors and policies, systems, and environments to improve dietary quality among rural, Native American residents [39].

### Individual factors

Understanding perceptions about healthy diets and the community food environment can inform contextually-appropriate approaches to improve consumer demand for healthy foods and beverages and aid in transforming larger policies, systems, and environments to support improved dietary quality [39, 40]. Participants at times had misinformed ideas about what items comprised a ‘healthy diet’ (e.g., whole grains or fruits perceived as ‘unhealthy’), which may in part lead to poor dietary quality experienced among this community [41]. However, participants shared major resource barriers to exercising optimal choices, such as time and money, that underscore the need for multiple disciplinary public health approaches to improve wages under reasonable working hours in addition to improving access to foods and beverages aligned with the 2015–2020 DGA.

### Community systems and environments

Environmental approaches to health promotion have been integral for working toward improving dietary quality among minority and Native American communities as a way to mobilize community strengths and reclaim traditional ways of knowing and food acquisition [39, 40, 42]. Among Flathead Reservation participants, both natural and built environmental sources of food were part of the community food supply. Social networks among families and community members were described as necessary for improving access to enough foods amidst barriers, including transportation. Food environment changes could be initiated based on these perceptions. The social nature of food access (e.g., family and community helping others secure foods) mirrors experiences among other rural and urban US consumers who are establishing non-traditional markets to buffer reduced access to healthy foods and beverages [43, 44]. Hollis-Hansen et al. (2019) found mobile produce markets a promising strategy for food security among low-income communities in comparison new supermarket introductions [45]. Mobile markets may be a potential food system strategy to connect home and community gardens to food insecure residents in affordable and accessible (e.g., transport) ways. Opportunities for tribal resources to support the delivery of nutrition cooking, and gardening education alongside food delivery could help increase consumer demand for produce deliveries [40]. Such approaches require testing to inform impact on household economies and dietary quality in Native American communities.

Participants in this study used grocery formats for the majority of household food needs, which mirrors current shopping patterns in the US [46]. Existing built environment resources (e.g., supermarkets and nontraditional stores) could be leveraged to encourage holistically improved dietary quality in this community. Healthy retail programs for example are utilized by the USDA SNAP-Education [47] and community partnerships with local SNAP-Ed agents could assist community store owners with changes that ensure healthy choices are more convenient than unhealthy products [48, 49]. Healthy retail programming could also target restaurants in this community due to participants’ time constraints and reliance on food away from home sources, and the effectiveness of co-approaches require more research [50], especially among Native American communities [40, 50, 51].

### Policies

Residents in this community rely on federal nutrition assistance programs for food security. While perceptions of various programs available in the community were favorable, some participants linked the history of reservations and government sourcing of food to poor health outcomes of Native peoples. Some of those included in this study seemed to have a mistrust of modern food systems (e.g., processing and large-scale growing practices) that may be compounded by a historical mistrust of the US government [52]. It could be argued that the impact of historical traumas with regard to Native food systems demand a higher accountability of government sourced foods in ensuring foods and beverages aligned with the 2015–2020 DGA are available, affordable, convenient, and desirable [16] among Native American residents. Strategies to test food SNAP/WIC package allotments that include compensation for the time cost of purchasing and preparing healthy foods [53] and/or strategies to align assistance programming with local community preferences are also needed.

### Recommendations

Based on findings from this study, the research team along with community partners is co-designing, implementing, and evaluating food and nutrition education interventions. The study team recommends the following recommendations for enhancing community food environments and supporting food security and dietary quality on the Flathead Indian Reservation. These recommendations may be applicable to other Indigenous and rural communities.

1. Food environment interventions in Indigenous communities should examine both the natural and built food environment as a contextually-appropriate approach toward improving dietary quality, mobilizing community strengths, and reclaiming traditional ways of knowing and food acquisition. Researchers and practitioners should utilize appropriate methodologies for evaluating the natural and built food environment following a food environment typology framework [54].
2. Food and nutrition interventions in Indigenous communities should focus on building skills and capacity for procuring food from the natural food environment including efforts to revitalize traditional ecological knowledge of wild foods and building capacity for cultivating hard-to access produce through home gardens.
3. Food and nutrition interventions should be delivered in ways that are culturally aligned with local ways of acquiring knowledge. In this study, we found that familial relationships and social networks are important ways of acquiring knowledge. Thus, food and nutrition interventions should focus at the household level and encourage social networking among families and community members including through popular social media avenues, which include short cooking or gardening demonstrations that meet participants’ ways for acquiring knowledge.
4. Interventions in Indigenous communities should acknowledge the dynamic nature of food environments and diets as well as community goals. Community members, practitioners, and researchers should incorporate current community goals for their food environments, which should acknowledge previous food environments and dietary patterns, but may or may not look similar to past depending upon collaborative aims.
5. Food environment and dietary interventions should be co-designed in collaboration with community members to incorporate community needs and desires as well as ensure the interventions are context specific and culturally and ecologically relevant.

### Strengths and limitations

A main strength of this research is the large sample size that is unusual for qualitative research and the focus on understanding perceptions of food environments among Native American communities. Social desirability bias is a possibility in dietary research. Future research could rephrase the questions to address the community at large versus the individual. For example, researchers could ask more questions to participants about the food practices of others in the community versus their own to decrease bias, which this investigation did in part. This study collected the perceptions of tribal and non-tribal residents. When qualitative results of tribal and non-tribal residents were compared by researchers, we did not observe major differences in the responses between participants based upon demographics. The authors hypothesize this finding is because of the broad question set that was designed to capture preliminary perceptions about the food environment, which participants share across the Flathead Reservation, and their own diet, which is influenced by the food environment. Future research should build upon these findings to tailor questions to elicit responses based upon specific demographics towards strengthening a particular intervention design, the food environment, and diets. Food consumption of Native American individuals is generally linked to diabetes and heart disease in the US, but it is important to capture the nuance of each Tribe’s food environment to address the specific food environment. Results are not directly transferable to other communities’ or Native American Tribes’ but may inform work in other Native communities. For example, difference in perception of the food environment and diets may be different across tribal and non-tribal residents in a different Native community, even though they were not in this study. However, the approach used could be useful in expanding the current state of knowledge surrounding food environment perceptions in a variety of communities.

## Conclusion

High diet-related non-communicable disease rates in Native American populations in addition to a multitude of individual and community health inequities demonstrate a need to transform food environments to create opportunities for healthy and culturally appropriate foods. To achieve improved dietary quality and food security among Native communities, changes among multiple sectors are required. Community-specific knowledge and perceptions are integral to realizing these goals and initiating sustainable changes. Among residents of the Flathead Reservation, nutrition education and skill-building could be enhanced with opportunities to reform local food systems to meet income and time constraints, due to caring for families and employment. Policy changes and support from tribal and academic partners are required to define and realize success.

## Data Availability

Data and materials are secured by the Flathead Nation. Please contact corresponding author to relay any requests.

## Abbreviations

US: United States
FDPIR: Federal distribution program on indian reservations
CBPR: Community-based participatory research
CSKT: Flathead Reservation of the Confederated Salish & Kootenai Tribes
DGA: Dietary guidelines for Americans, 2015–2020
WIC: Special supplemental nutrition assistance program for women, infants, and children
